# Efficacy and Safety of Cryotherapy Combined With Intralesional Steroid for Keloid and Hypertrophic Scar: A Systematic Review and Meta‐Analysis

**DOI:** 10.1111/jocd.70924

**Published:** 2026-05-13

**Authors:** Xiaowei Feng, Xingwei Zou, Shali Jiang, Xin He, Yongqiong Deng, Jinwei Xie

**Affiliations:** ^1^ Department of Dermatovenerology Chengdu Integrated TCM& Western Medicine Hospital Chengdu China; ^2^ Department of Orthopedics Surgery West China Hospital of Sichuan University Chengdu China

**Keywords:** cryotherapy, hypertrophic scar, intralesional steroid, keloid, meta‐analysis

## Abstract

**Objective:**

Intralesional steroid injection is the first‐line therapy for scars. Emerging evidence indicates that combining intralesional steroid injection with cryotherapy yields promising therapeutic outcomes for scar management. However, a consensus on the efficacy and safety of this combined regimen has not yet been reached. Consequently, the present meta‐analysis aimed to assess the efficacy and safety of this combination compared with control groups.

**Methods:**

A full‐scale literature retrieval was performed across a range of databases to identify eligible randomized controlled trials (RCTs) and prospective non‐randomized studies that evaluated the efficacy and safety of cryotherapy plus intralesional steroid injection against control treatments for keloids or hypertrophic scars. Key outcome data, including the mean percentage change in scar improvement, the excellent clinical response rate (defined as ≥ 75% reduction in scar volume), and the incidence of adverse events, were extracted. Meta‐analyses were performed using a fixed‐ or random‐effects model based on the heterogeneity assessment results.

**Results:**

Thirteen studies involving 1012 cases were included in this meta‐analysis. The pooled results exhibited that cryotherapy combined with intralesional steroid injection yielded a significantly higher efficacy rate than various control interventions (including intralesional steroid monotherapy, cryotherapy alone, bleomycin/verapamil treatment, laser plus steroid, and surgical excision plus radiotherapy) (risk ratio [RR] = 1.19, 95% confidence interval (CI): 1.03–1.36; *p* = 0.01). However, no statistically significant differences were detected between the experimental and control groups in either the mean percentage change in scar improvement (standard mean difference (SMD) = 0.14, 95% CI: −0.39 to 0.68; *p* = 0.60) or the overall incidence of adverse events (RR = 1.35, 95% CI: 0.99–1.83; *p* = 0.06).

**Conclusions:**

Cryotherapy combined with intralesional steroid injection is a viable alternative to other first‐line treatments for keloids and hypertrophic scars. Clinical recommendations should be made cautiously based on specific patient and scar conditions. Further well‐designed, large‐scale RCTs are required to validate the long‐term efficacy and safety of this combined regimen.

## 1 Introduction

Keloids and hypertrophic scars refer to benign hyperplasia of dense fibrous tissue arising from an abnormal reparative reaction to a variety of external or internal stimuli, including skin trauma, burns, surgeries, injections, and chronic inflammation [1]. Keloids and hypertrophic scars may develop due to an imbalance between collagen synthesis and degradation during the remodeling phase. These lesions tend to occur in individuals with darker skin tones and are associated with increased skin surface tension, genetic susceptibility, and hormonal factors [2]. Notably, although keloids are non‐fatal, they often cause cosmetic disfigurement and symptoms of pruritus or pain [3].

Various therapeutic modalities are currently used to manage keloids and hypertrophic scars, including surgery, radiotherapy, intralesional injections of corticosteroids, bleomycin, or 5‐fluorouracil (5‐Fu), cryotherapy, laser therapy (with or without intralesional steroid injection), silicone gel application, and compression therapy [4]. However, the multiplicity of treatment methods indicates that none of these modalities is entirely satisfactory. Consequently, clinicians are continually exploring optimized therapeutic strategies to achieve higher cure rates, lower adverse‐effect incidences, and reduced recurrence rates.

Among nonsurgical modalities, intralesional corticosteroid injection is regarded as the first‐line therapeutic option for the management of keloids or hypertrophic scars [5]. Corticosteroids, including triamcinolone acetonide (TAC) and betamethasone, exert therapeutic effects by reducing collagen synthesis, increasing glycosaminoglycan deposition, suppressing fibroblast proliferation, and inducing local hypoxia, thereby attenuating the inflammatory cascade [6].

Cryotherapy involves applying extreme cold (typically using liquid nitrogen at −196°C) to treat many skin conditions, including keloids [7]. Local cryotherapy with liquid nitrogen exerts direct cytotoxic effects on keloid tissues, modulates collagen synthesis pathways, and further induces the phenotypic normalization of keloidal fibroblasts [8]. Emerging evidence suggests that cryotherapy pre‐treatment can induce localized edema, enhancing the permeation of subsequently injected corticosteroids and optimizing their intralesional distribution, potentially yielding superior clinical outcomes. However, a unified consensus regarding the efficacy and optimal protocol of this combined strategy has not yet been established in existing literature.

Against this backdrop, the present meta‐analysis was conducted to systematically evaluate the therapeutic efficacy and safety of cryotherapy combined with intralesional corticosteroid injection compared with alternative monotherapies or other combined regimens for managing keloids and hypertrophic scars.

## 2 Materials and Methods

### 2.1 Data Sources and Search Strategy

In adherence to the PRISMA guidelines [9], an exhaustive literature search was undertaken across four major electronic databases, namely PubMed, EMBASE, the Cochrane Central Register of Controlled Trials, and Web of Science. The search covered all publications from the inception of each database to January 1, 2026. The key search terms included “Keloid,” “Hypertrophic scar,” “Corticosteroid,” “Triamcinolone,” “Intralesional injection,” “cryotherapy,” and “liquid nitrogen,” with no restrictions placed on the language of publication. Furthermore, the reference lists of all included studies were manually reviewed to identify additional eligible trials. Study selection and assessment were conducted independently by two reviewers to minimize selection bias.

### 2.2 Inclusion and Exclusion Criteria

Eligibility criteria were defined a priori as follows. Inclusion criteria: (1) Study design was restricted to randomized controlled trials (RCTs) and prospective controlled clinical trials (CCTs), with no constraints on allocation concealment or blinding procedures; (2) Participants were diagnosed with keloids or hypertrophic scars; (3) Interventions involved combined cryotherapy and intralesional corticosteroid injection, in comparison with alternative therapeutic modalities; (4) Outcome measures included excellent response rate, mean percentage changes in scar volume, height and thickness, patient‐reported outcomes and observer‐assessed scores, Vancouver Scar Scale (VSS) scores pre‐ and post‐treatment, recurrence rate, and adverse events. Exclusion criteria: (1) Preclinical studies involving animal models or in vitro cell experiments; (2) Non‐RCT and non‐CCT study designs; (3) Studies that did not adopt combined therapeutic strategies for keloids or hypertrophic scars; (4) Studies with insufficiently reported outcomes or those irrelevant to the objectives of the present meta‐analysis [10].

### 2.3 Data Extraction and Quality Assessment

Two reviewers independently extracted data from eligible studies, with extracted information covering the first author, year of publication, sample sizes of the experimental and control groups, patient baseline characteristics (e.g., age, sex), detailed descriptions of interventions and comparators, the number of treatment cycles, and follow‐up duration. The outcomes of interest in this meta‐analysis included the excellent clinical response rate, mean percentage of scar improvement post‐treatment, recurrence rate, and adverse event rate. Data extraction was performed using a pre‐designed data collection form in accordance with the intention‐to‐treat principle. Study quality assessment was conducted independently by two reviewers: the Cochrane Risk of Bias Tool (from the Cochrane Handbook for Systematic Reviews of Interventions) was applied to evaluate eligible randomized controlled trials, while the ROBINS‐I tool was used for non‐randomized studies [11]. Any discrepancies arising during the assessment process were resolved through consultation with a third reviewer to achieve a final consensus.

### 2.4 Statistical Analysis

This systematic review and meta‐analysis was performed using Review Manager software (RevMan, version 5.4; The Cochrane Collaboration, 2020). Data preprocessing was conducted with Microsoft Excel. For continuous outcomes, namely the mean percentage change in clinical indicators, the standard mean difference (SMD) with 95% confidence intervals (CIs) was used for pooled analysis. For dichotomous outcomes including the excellent clinical response rate and adverse event incidence, risk ratios (RRs) with 95% CIs were calculated. Statistical heterogeneity across included studies was assessed via the chi‐squared test and the *I* ^2^ statistic, with heterogeneity considered significant if *I* ^2^ > 50% or *p* < 0.10. A fixed‐effects model was employed to pool effect sizes in the absence of significant heterogeneity; otherwise, a random‐effects model was utilized. Subgroup analyses were stratified by the type of control intervention and adverse event category. Sensitivity analysis was conducted by sequentially excluding each individual study to determine its impact on the overall pooled results. Publication bias was evaluated using funnel plots, and statistical significance was set at *p* < 0.05.

## 3 Results

### 3.1 Data Extraction and Study Characteristics

**FIGURE 1:**
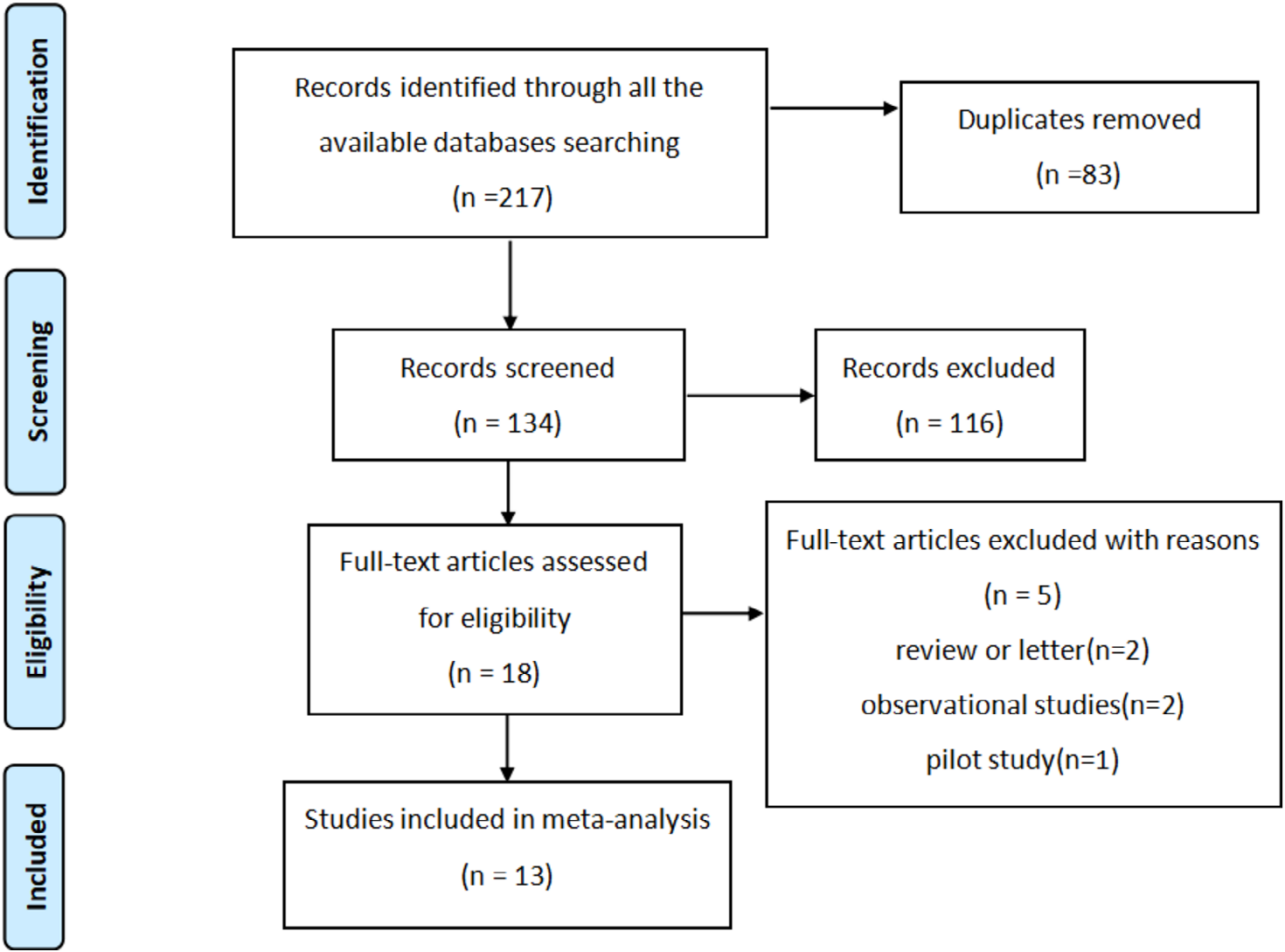
Flowchart of the study selection process.

A total of 217 studies were retrieved initially from the Cochrane Library, Embase, PubMed, and Web of Science databases. Following the removal of duplicate records (*n* = 83), the remaining 134 studies underwent title and abstract screening, with 18 studies deemed eligible for full‐text assessment. Subsequent full‐text review led to the exclusion of an additional 5 studies, consisting of 1 pilot study, 2 observational studies, and 2 narrative reviews. Eventually, 13 articles published from 2001 to 2025 [12, 13, 14, 15, 16, 17, 18, 19, 20, 21, 22, 23, 24] were included in the present meta‐analysis (Figure 1). Comprehensive characteristics of the included studies are presented in Table 1.

**TABLE 1:** Characteristics of included studies.

Study (country)	Age (Y)	Gender (F/M)	Study design	Sample (E/C)	Interventions (E)	Interventions (C)	Treatment duration	Follow‐up	Measures
Abbasi 2025 [12] (Pakistan)	18–60	25/51	RCT	38/38	Cryotherapy + intralesional TAC (40 mg/mL)	Intralesional TAC alone	3 times at 4‐weekly intervals	12 weeks	Vancouver Scar Scale (VSS), Patient Scar Assessment Scale (PSAS), Treatment efficay (> 75% reduction in VSS)
Ahsan 2018 [13] (Bangladesh)	> 12	N/A	RCT	30/30	Cryotherapy + intralesional TAC	Intralesional TAC alone	At least 3 sessions spaced 4 weeks apart	12 weeks	Recovery rate, clinical response
Cohen 2022 [15] (USA)	13–79	86/23	Prospective control study	34/75	Cryotherapy + intralesional TAC	Different concentrations of TAC alone	N/A	N/A	Pruritus score, pain score, changes in size
Behera 2016 [14] (India)	> 12	N/A	RCT	30/30	Cryotherapy at baseline + Intralesional TAC (40 mg/mL)	Carbon dioxide laser + Intralesional TAC	4 weeks interval for 3 months	3 months, 6 months, 12 months	Therapeutic responses, reduction in volume, patient self‐assessment (PSA) score, blinded observer assessment score (OA), VSS, side effects, recurrence
Meymandi 2016 [19] (Iran)	Mostly 21–40	120/46	RCT	83/83	Cryotherapy + intralesional TAC	Intense pulsed light + Intralesional TAC	3‐week interval with a maximum of 8 sessions	N/A	VSS, recovery rate, side effects, Improvement of lesions, treatment sessions, satisfaction
Nishi 2022 [21] (India)	Mostly 21–50	80/90	RCT	85/85	Cryotherapy + intralesional TAC	Fractional CO_2_ laser + Topical betamethasone	4 sessions at 4‐week interval	4 months	Modified manchester scar scale score (MSS), pain assessment, side effects
Emad 2010 [16] (Iran)	16–45	13/13	Prospective control study	9/19	Cryotherapy + intralesional TAC	Surgical excision and radiotherapy	Multiple sessions every 20 days till complete flattening	at least 1 year	Rate of complete remission, patients' self‐assessment of treatment, side effects, treatment sessions, reccurence
Jannati 2015 [17] (Iran)	10–50	42/38	RCT	20/20/20/20	Cryotherapy + intralesional TAC	Intralesional verapamil + cryotherapy/Intralesional verapamil/Cryotherapy alone	3‐week interval with a maximum of 8 sessions	52 weeks	VSS, outcomes of treatment, side effects
Krishna 2025 [18] (India)	18–51	42/48	RCT	30/30/30	Cryotherapy+intralesional TAC	Intralesional bleomycin + TAC/Fractional CO_2_ laser	4 sessions at 4‐week interval	8 weeks, 16 weeks, 6 months	VSS, improvement in skin thickness, adverse effects, recurrence
Naeini 2006 [20] (Iran)	13–55	31/14	Prospective control study	23/22	Cryotherapy + intralesional TAC	Bleomycin tattoo	4 sessions at 4‐week interval	3 months	Therapeutic responses, side effects, disappearance of symptoms, recurrence
Sharma2007 [22] (India)	N/A	8/13	RCT	30/30	Cryotherapy + intralesional TAC	Cryotherapy alone	1 month interval for a maximum of 6 months	18 months	Excellent response, recurrence
Yosipovitch 2001 [23] (Singapore)	17–48	1/9	Prospective control study	10/8/10	Cryotherapy + intralesional TAC	Cryotherapy alone/TAC injection alone	3 times at 4‐weekly intervals	8 months	Thickness change, pain, itch, side effects, recurrence
Zouboulis 2020 [24] (Greece)	14–44	22/18	RCT	20/20	Cryotherapy + intralesional betamethasone	Cryotherapy alone	3 monthly sessions	6–36 months	Lesional volume change, excellent response, adverse effects, histological and immunohistological changes

Abbreviations: C, control group; E, experimental group; F, female; M, male; N/A, not applicable; RCT, randomized controlled trial; TAC, triamcinolone acetonide; Y, years.

### 3.2 Risk of Bias

**FIGURE 2:**
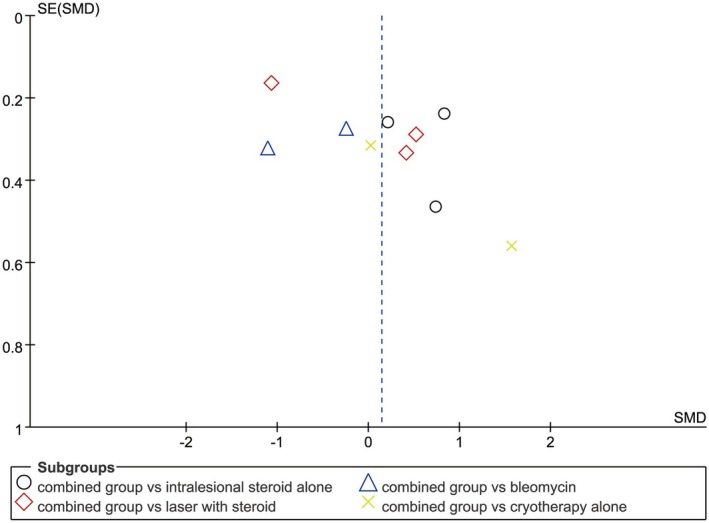
Funnel plot of bias risk.

Tables 2 and 3 summarize the methodological quality and bias risk of the included studies. All nine randomized clinical trials were relatively well‐designed (modified Jadad scale score > 4). The ROBINS‐I tool was applied to evaluate the risk of bias across non‐randomized studies. Among these studies, three were classified as having low overall risk of bias, and only one was rated as moderate risk, thus demonstrating the high quality of the included literature. The funnel plot presented a symmetrical distribution of all included trials around the pooled standard error of the mean difference (MD), which suggested a low likelihood of publication bias in this meta‐analysis (Figure 2).

**TABLE 2:** Quality assessment of randomized controlled studies according to the Cochrane Risk of Bias Tool.

Studies	Random sequence generation	Allocation concealment	Blinding	Incomplete outcome data	Selective reporting	Other bias	Jadad score (0–8)
Abbasi 2025	Low	Low	Unclear	Low	Low	Low	7
Ahsan 2018	Low	Low	Low	Low	Low	Low	8
Behera 2016	Low	Low	Low	Low	Low	Low	8
Meymandi 2016	High	Low	Low	Low	Low	Low	7
Nishi 2022	Low	Unclear	Unclear	Low	Low	Low	7
Jannati 2015	Low	Low	Low	Low	Low	Low	8
Krishna 2025	Low	Low	High	Low	Low	Low	7
Sharma 2007	Low	High	Unclear	Low	Low	Low	7
Zouboulis 2020	Low	Low	Low	Low	Low	Low	8

*Note:* Unclear, lack of information or uncertainty over the potential bias.

**TABLE 3:** Risk of bias of non‐randomized studies according to the ROBINS‐I tool.

Studies	Bias due to confounding	Bias in the selection of participants into the study	Bias in the classification of interventions	Bias due to deviations from intended interventions	Bias due to missing data	Bias in measurement of the outcomes	Bias in selection of the reported results	Overall risk of bias
Cohen 2022	Moderate	Low	Low	Moderate	Low	Low	Moderate	Moderate
Emad 2010	Low	Low	Low	Low	Moderate	Low	Low	Low
Naeini 2006	Low	Moderate	Low	Low	Low	Low	Low	Low
Yosipovitch 2001	Low	Low	Low	Low	Low	Low	Moderate	Low

### 3.3 Effects of Cryotherapy Plus Intralesional Steroid Compared With the Control Groups

#### 3.3.1 Excellent Clinical Response Rate

**FIGURE 3:**
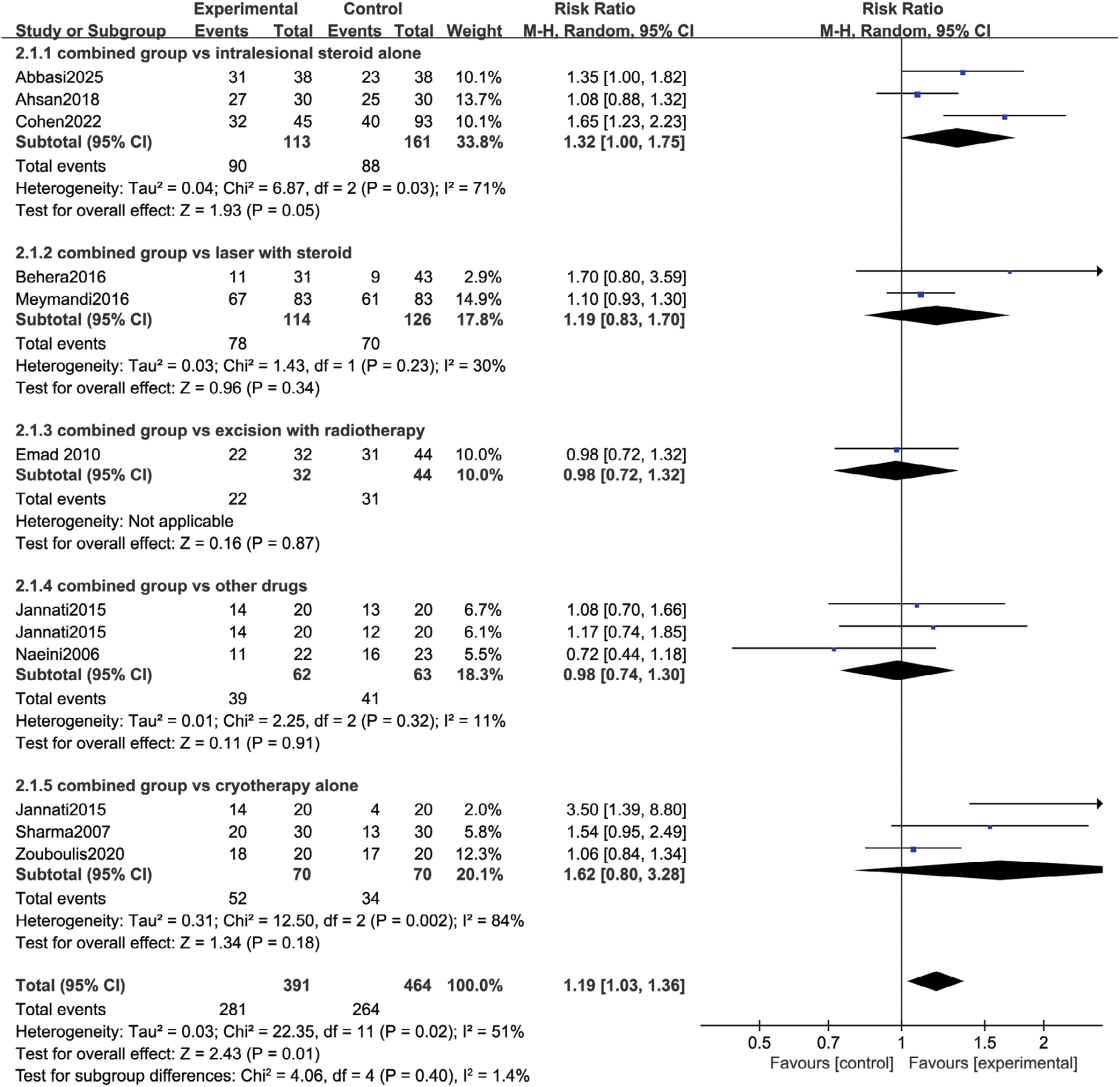
Forest plot of excellent clinical response rate at the end.

The meta‐analysis comparing the excellent response rate between the cryotherapy combined with intralesional steroid and the control groups exhibited that the experimental combined group achieved a significantly higher rate than the control groups (RR = 1.19, 95% CI: 1.03–1.36; *p* = 0.01) with moderate heterogeneity (*I* ^2^ = 51%, *p* = 0.02) (Figure 3). Subgroup analysis was conducted by treatment method in the control groups, as these differences might have introduced bias and distorted the synthesis and analysis of results.

##### 3.3.1.1 Cryotherapy Combined With Intralesional Steroid Against Intralesional Steroid Alone

Three RCTs [12, 13, 15] evaluated the efficacy of the combined group compared with intralesional steroid monotherapy. A total of 113 cases in the experimental group and 161 cases in the control group were included. Given the presence of moderate heterogeneity (*I* ^2^ = 71%, *p* = 0.03), a random‐effects model was used for statistical analysis. The efficacy rate was marginally significantly different between the two groups (RR = 1.32, 95% CI: 1.00–1.75, *p* = 0.05), indicating that the combined intervention group had a slightly higher efficacy rate than the steroid monotherapy group.

##### 3.3.1.2 Cryotherapy Combined With Intralesional Steroid Versus Laser With Intralesional Steroid

Two studies [14, 19], comprising 114 and 126 cases in the experimental and control groups, respectively, were included. The results demonstrated no statistically significant difference between the two groups (RR = 1.19, 95% CI: 0.83–1.70; *p* = 0.34). Low heterogeneity was noted (*I* ^2^ = 30%, *p* = 0.23), suggesting consistent effects across the studies.

##### 3.3.1.3 Cryotherapy Combined With Intralesional Steroid Against Excision With Radiotherapy

Emad et al. [16] evaluated the efficacy and safety of the combined treatment against surgery plus radiotherapy. 32 and 44 patients were allocated to the experimental and excision groups, respectively. The results indicated no efficacy difference between the two groups (RR = 0.98, 95% CI: 0.72–1.32; *p* = 0.87).

##### 3.3.1.4 Cryotherapy Combined With Intralesional Steroid Against Other Drugs

Two studies [17, 20] compared the effectiveness of the combined group (TAC plus cryotherapy) with that of the control groups treated with bleomycin or verapamil (with or without cryotherapy). The results exhibited that the efficacy rate of patients treated with steroids plus cryotherapy was not significantly higher than that of patients treated with the drugs (RR = 0.98, 95% CI: 0.74–1.30; *p* = 0.91), with low heterogeneity (*I* ^2^ = 11%, *p* = 0.32).

##### 3.3.1.5 Cryotherapy Combined With Intralesional Steroid Against Cryotherapy Alone

Three RCTs [17, 22, 24] were included, involving 70 cases in both the experimental group and the control group. When comparing the combined group with the cryotherapy‐alone group, there was no statistically significant difference in efficacy rate (RR = 1.62, 95% CI: 0.80–3.28; *p* = 0.18), with high heterogeneity observed (*I* ^2^ = 84%, *p* = 0.002).

#### 3.3.2 Mean Percentage of Change in Clinical Indicators

**FIGURE 4:**
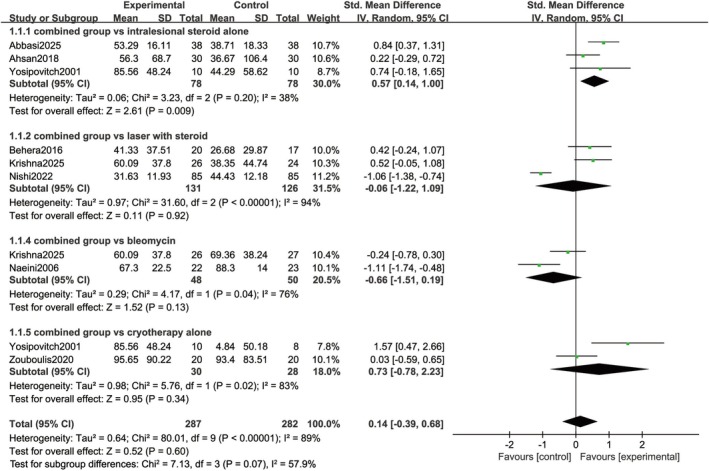
Forest plot of the mean percentage of change in scar improvement.

The present forest plot (Figure 4) was constructed to assess the mean percentage change in predefined primary outcomes, including VSS scores, scar volume, or height reduction, from baseline to the post‐treatment follow‐up period, with comparisons between the experimental group and four distinct control subgroups. A random‐effects model was employed for data pooling, given the anticipated inter‐study variability. The results demonstrated a non‐significant between‐group difference in outcome changes (SMD = 0.14, 95% CI: −0.39–0.68; *p* = 0.60), with extreme statistical heterogeneity (*I* ^2^ = 89%, *p* < 0.00001). Sensitivity analyses were conducted by study type to address substantial heterogeneity. The results demonstrated that the pooled effect remained robust and consistent sequentially excluding any individual study. Specifically, the pooled effect yielded an SMD of −0.05 (95% CI: −0.62–0.51; *p* = 0.85) after omitting Yosipovitch [23], and an SMD of 0.28 (95% CI: −0.27–0.83; *p* = 0.32) after excluding Naeini [20]. These findings confirm that no single study exerted an undue influence on the overall meta‐analytic results.

##### 3.3.2.1 Cryotherapy Combined With Intralesional Steroid Against Intralesional Steroid Alone

A total of three studies [12, 13, 23] were included in the subgroup analysis. The pooled results demonstrated that the combined intervention group exhibited a statistically significant improvement in the primary outcome compared to the control group (SMD = 0.57, 95% CI: 0.14–1.00; *p* = 0.009). Meanwhile, heterogeneity assessment indicated relatively low inter‐study heterogeneity (*I* ^2^ = 38%, *p* = 0.20), suggesting good consistency in the intervention effect across the three included studies.

##### 3.3.2.2 Cryotherapy Combined With Intralesional Steroid Against Laser With Intralesional Steroid

Three studies [14, 18, 21] were included in this subgroup. Pooled results revealed no statistically significant between‐group differences (SMD = −0.06, 95% CI: −1.22–1.09; *p* = 0.92), whereas extreme inter‐study heterogeneity was observed (*I* ^2^ = 94%, *p* < 0.00001). This high heterogeneity was largely attributed to the study by Nishii et al. [21], which reported a directionally opposite (negative) intervention effect.

##### 3.3.2.3 Cryotherapy Combined With Intralesional Steroid Against Bleomycin

Two studies [18, 20] contributed to this subgroup. The pooled results exhibited no statistically significant between‐group differences (SMD = −0.66, 95% CI: −1.51–0.19; *p* = 0.13). High inter‐study heterogeneity was detected (*I* ^2^ = 76%, *p* = 0.04), which was attributable to the relatively large negative intervention effect reported by Naeini [20].

##### 3.3.2.4 Cryotherapy Combined With Intralesional Steroid Against Cryotherapy Alone

This subgroup included two eligible studies [23, 24]. The pooled results indicated no statistically significant between‐group differences (SMD = 0.73, 95% CI: −0.78–2.23; *p* = 0.34) in the random‐effects model. High inter‐study heterogeneity was observed (*I* ^2^ = 83%, *p* = 0.02), which was driven by the relatively large positive intervention effect reported by Yosipovitch [23].

### 3.4 Adverse Events and Recurrence

All studies included in this meta‐analysis reported the occurrence of adverse events after therapeutic interventions for keloids or hypertrophic scars. A pooled analysis of overall adverse event incidence was conducted, comparing the cryotherapy combined with intralesional steroid injection and the control groups. The results demonstrated no statistically significant difference in the risk of side effects between the two cohorts (RR = 1.35, 95% CI: 0.99–1.83; *p* = 0.06), with moderate heterogeneity detected across the included studies (*I* ^2^ = 50%, *p* = 0.0008).

**FIGURE 5:**
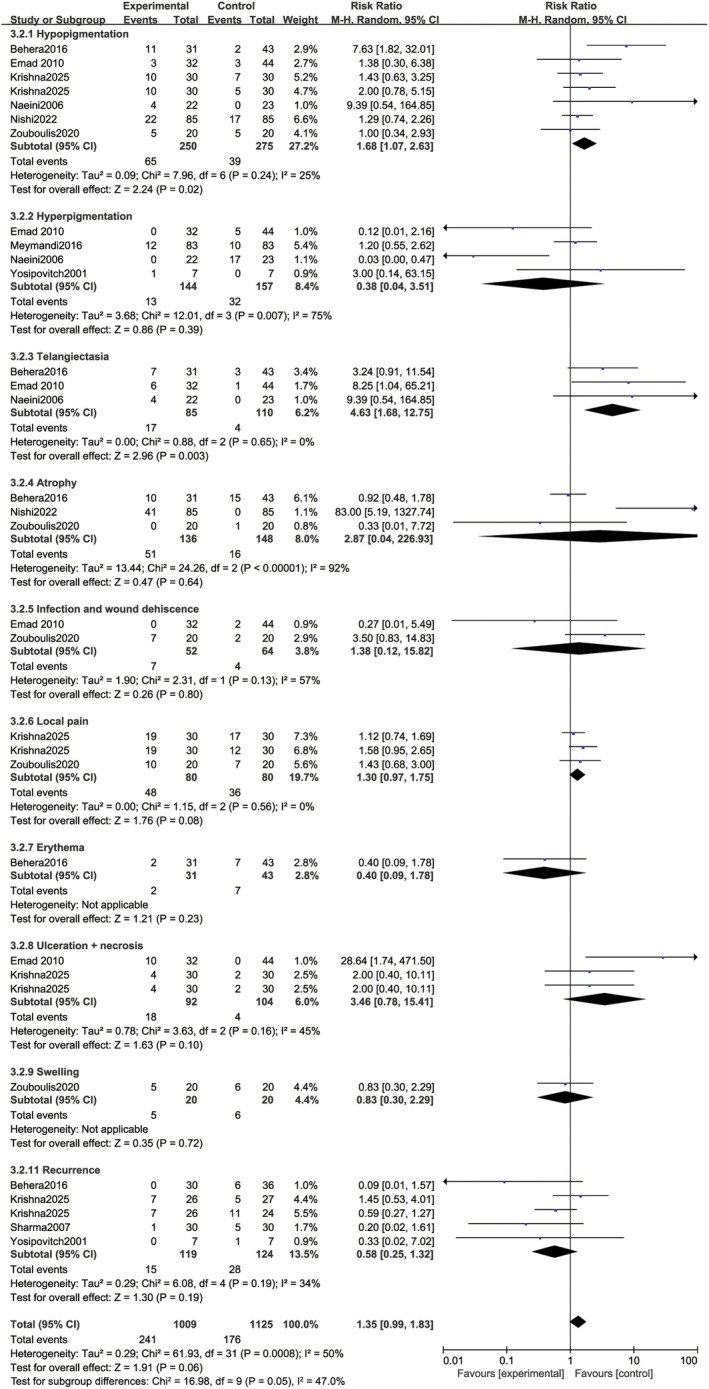
Forest plot of side effects.

Subgroup analyses were performed for individual adverse events to further explore the safety profile of the combined intervention, and the results are presented in Figure 5. Notably, the combined treatment group exhibited a significantly elevated risk of hypopigmentation (RR = 1.68, 95% CI: 1.07–2.63; *p* = 0.02) and telangiectasia (RR = 4.63, 95% CI: 1.68–12.75; *p* = 0.003) than the control group. Conversely, no statistically significant between‐group differences were identified for the remaining adverse events, including hyperpigmentation (RR = 0.38, 95% CI: 0.04–3.51; *p* = 0.39), cutaneous atrophy (RR = 2.87, 95% CI: 0.04–226.93; *p* = 0.64), infection or wound dehiscence (RR = 1.38, 95% CI: 0.12–15.82; *p* = 0.80), local pain (RR = 1.30, 95% CI: 0.97–1.75; *p* = 0.08), erythema (RR = 0.40, 95% CI: 0.09–1.78; *p* = 0.23), ulceration complicated with necrosis (RR = 3.46, 95% CI: 0.78–15.41; *p* = 0.10), and local swelling (RR = 0.83, 95% CI: 0.30–2.29; *p* = 0.72). Moderate heterogeneity was consistently observed across these subgroup analyses (*I* ^2^ = 50%, *p* = 0.0008).

Regarding post‐treatment recurrence of keloids or hypertrophic scars, relevant data were extracted from four eligible studies [14, 18, 22, 23]. Meta‐analytical results indicated that the combined intervention group exhibited a numerically lower recurrence rate than the control groups, but this trend did not reach statistical significance (RR = 0.58, 95% CI: 0.25–1.32; *p* = 0.19). Additionally, low inter‐study heterogeneity was noted for this outcome (*I* ^2^ = 34%, *p* = 0.19), suggesting good consistency in recurrence‐related findings across the included studies.

## 4 Discussion

Hypertrophic scars and keloids are common benign fibrous hyperplastic lesions in dermatological clinics [25]. Characterized by excessive collagen deposition beyond the original wound boundaries, these lesions not only cause cosmetic concerns but also lead to functional limitations, including itching, pain, and joint contracture in severe cases, thereby imposing significant burdens on both clinicians and patients [26]. Although various treatment modalities, including surgical excision, laser therapy, intralesional steroids, or cryotherapy, have been applied in clinical practice, single‐agent therapies are often limited by suboptimal therapeutic efficacy and unacceptably high recurrence rates. Against this backdrop, combination treatment regimens have been increasingly explored and have gradually emerged as promising strategies for keloid management [27]. Among these, cryotherapy combined with intralesional steroid injection is one of the most used and clinically effective options for hypertrophic scars and keloids. However, discrepancies in study design, patient populations, and outcome assessment metrics across existing clinical studies have led to inconsistent findings and no unified consensus on the efficacy and safety of this combined therapy to date. To fill this critical knowledge gap, the present meta‐analysis is the first to systematically and comprehensively compare the therapeutic efficacy and safety of cryotherapy combined with intralesional steroid injection with those of other routine treatment modalities for hypertrophic scars and keloids.

Glucocorticoids exert therapeutic effects on hypertrophic scars and keloids through multiple synergistic mechanisms. First, they directly inhibit the proliferation of dermal fibroblasts and suppress the synthesis and deposition of extracellular matrix components, particularly collagen, which is a key driver of fibrous hyperplasia in scar lesions. Second, they modulate the local cytokine microenvironment by downregulating pro‐fibrotic cytokine expression and upregulating anti‐fibrotic factors, thereby disrupting the pro‐fibrotic cascade. Third, they exert potent anti‐inflammatory effects by inhibiting the infiltration and activation of inflammatory cells at the scar site, helping resolve the chronic inflammatory state that sustains scar progression. Additionally, glucocorticoids can reduce the vascularity of hypertrophic scars and keloids via anti‐angiogenic effects, impairing the nutrient supply to hyperplastic scar tissue, alleviating tissue hyperplasia, and promoting scar maturation [28]. Despite these beneficial effects, monotherapy with intralesional corticosteroid injections has inherent limitations: It can only soften and flatten keloid lesions to a certain extent and cannot achieve complete regression of keloids or effectively narrow the width of wide hypertrophic scars [29]. This therapeutic gap highlights the rationale for combining glucocorticoid injections with other modalities, such as cryotherapy, in clinical practice.

Cryosurgery is a pivotal therapeutic modality for various cutaneous proliferative disorders, including hypertrophic scars and keloids. Its core mechanism involved rapid local cooling, inducing irreversible cellular damage and apoptosis in fibroblasts [30]. This extreme hypothermia disrupts intracellular organelles, denatures functional proteins, and impairs collagen synthesis, thereby inhibiting fibroblast proliferation and reducing scar tissue volume. Additionally, cryotherapy modulates the local inflammatory microenvironment by suppressing the secretion of pro‐fibrotic cytokines and inducing vascular endothelial cell damage, thereby further alleviating scar hyperplasia and reducing lesion vascularity [31]. In vitro studies have confirmed that cryotherapy can alter the collagen synthesis pattern and differentiation of keloid fibroblasts, shifting them toward a normal phenotype [32]. Despite these favorable effects, cryotherapy as monotherapy typically requires multiple treatment sessions to achieve satisfactory clinical outcomes.

The findings of the present meta‐analysis demonstrate that cryotherapy combined with intralesional steroid injection achieves a statistically significant superior efficacy rate compared with a range of monotherapeutic approaches for managing hypertrophic scars and keloids. Among all subgroup comparisons, the most notable therapeutic advantage was observed when the combined intervention was compared with intralesional steroid monotherapy. This result strongly supports the synergistic interaction between the two modalities. Two key mechanisms may underpin this synergism. First, cryotherapy‐induced disruption of the dense, fibrous scar matrix enhances the penetration of injected corticosteroids into the target tissue, thereby maximizing local drug bioavailability. Second, the dual anti‐angiogenic effects of the combination, cryotherapy‐mediated immediate vasoconstriction and steroid‐induced long‐term inhibition of angiogenesis, act in concert to reduce scar vascularity, impairing the nutrient supply required for fibroblast proliferation and scar hyperplasia [33]. Furthermore, precision cryotherapy mitigated the pain associated with intralesional steroid injections for keloids [34], a benefit that may improve patient compliance with repeated treatment sessions. Consistent with these observations, Zouboulis et al. [24] reported that cryosurgery alone and cryosurgery combined with intralesional corticosteroids are effective and well‐tolerated for the treatment of small keloids in young patients. The modality exerts therapeutic effects not only through direct physical destruction of hyperplastic scar tissue but also by initiating biochemical and immunological cascades that drive “scar rejuvenation,” thereby normalizing the pathological microenvironment of keloid lesions.

However, the present meta‐analysis revealed that combining cryotherapy with intralesional steroids did not confer a statistically significant efficacy advantage over a panel of alternative therapeutic modalities for keloid and hypertrophic scar management, namely surgical excision combined with radiotherapy, laser therapy plus intralesional steroids, bleomycin, and cryotherapy monotherapy. This lack of differential benefit can be primarily ascribed to the fact that all these comparator strategies are classified as first‐line keloid treatments, with comparable clinical potency in modulating fibrotic scar tissue. Among these alternative regimens, surgical excision stands out for its ability to achieve the most pronounced volumetric reduction of keloid lesions by physically removing hyperplastic scar tissues. However, the high inherent recurrence rate of excised keloids mandates adjuvant radiotherapy, suppresses residual fibroblast activity, and prevents lesion regrowth. A critical limitation of this combined surgical‐radiotherapeutic approach lies in the strict threshold of cumulative radiation dose that must not be exceeded to minimize the radiation‐induced adverse event risks. Consequently, this regimen is generally not amenable to retreatment in patients who experience keloid recurrence after initial therapy [35]. Laser therapy combined with intralesional corticosteroid injections has emerged as a well‐established and effective option for managing small‐to‐medium‐sized keloids. With pulsed dye lasers targeting scar vasculature and CO_2_ lasers facilitating collagen remodeling, this modality exhibits superior performance in improving scar color and texture, making it the preferred option for keloids in aesthetically sensitive areas [36]. However, its widespread use is constrained by its relatively high cost and the need for specialized training to ensure precise and safe treatment delivery, particularly in resource‐limited regions. Additionally, a growing body of clinical evidence has corroborated the therapeutic efficacy of several intralesional agents, including 5‐Fu, bleomycin, verapamil, and botulinum toxin A, in managing hypertrophic scars and keloids [37]. These agents exert distinct anti‐fibrotic effects through different mechanisms. To maximize therapeutic outcomes, these agents are almost exclusively administered in combination with other treatments. However, their translation into routine clinical practice is hindered by two major challenges: limited accessibility and the potential for drug‐related toxicities, necessitating careful patient selection and monitoring.

Another critical factor contributing to the suboptimal efficacy and high complication rates observed in the included trials is the exclusive adoption of spray or contact cryotherapy, two of the most widely used clinical cryosurgical techniques. However, these two approaches fail to achieve sufficient freezing depth within a short treatment duration and cannot effectively reach the lesion base, resulting in high recurrence rates [38]. Additionally, surface cryotherapy often leaves an open, exudative wound that is prone to infection and requires weeks to heal. Moreover, melanocytes are highly sensitive to low temperatures; therefore, this approach inevitably causes a certain degree of skin atrophy and prolonged hypopigmentation. These limitations render surface cryotherapy suboptimal for dark‐skinned patients [39]. Conversely, intralesional cryosurgery directly delivers maximum cold intensity to deeper tissue layers, immediately destroying the keloid core by targeting its cellular components and blood vessels, while minimizing damage to superficial tissues and melanocytes. This technique effectively reduces scar volume and alleviates associated symptoms with few adverse effects [40, 41, 42, 43]. However, it remains less effective than keloid excision combined with brachytherapy for managing refractory keloid lesions [44].

From a safety perspective, the combined cryotherapy‐intralesional steroid intervention was associated with a near‐significant increase in the overall risk of adverse events. Specifically, statistically significant increases were observed in the incidence of hypopigmentation and telangiectasia, complications that align with the established toxicities of the two modalities: Cryotherapy‐induced melanocyte damage underlies pigmentary disturbances, whereas steroid‐mediated vascular fragility contributes to telangiectasia development. Conversely, other complications, including cutaneous atrophy and ulceration, exhibited extreme inter‐study heterogeneity, likely stemming from variations in study populations, treatment protocols, and follow‐up durations across the included trials.

A subset of the study data was derived using statistical calculations. The observed discrepancy, a marginally significant between‐group difference in efficacy rate versus non‐significant differences in mean percentage change from baseline and adverse event rate, is consistent with the well‐documented challenges in outcome assessment for keloid treatment. The excellent clinical response rate (defined as ≥ 75% reduction in lesion size) is inherently sensitive to categorical response capture, even to minor improvements. The continuous endpoint of mean percentage change reflects the aggregate response of the entire cohort, diluting the impact of individual favorable outcomes. Supported by a non‐significant mean percentage change and adverse events that confirm limited overall therapeutic benefit, it reinforced that the efficacy rate difference is a minor statistical finding rather than a clinically impactful advantage.

Several limitations need to be addressed when interpreting the findings of this meta‐analysis. First, the absence of a unified control group across included studies, coupled with small sample sizes in key subgroups, reduced statistical power, increased the risk of type II errors, and limited the generalizability of the results. Second, substantial inter‐study heterogeneity may have masked true treatment effects; variability in scar etiologies, patient demographics, outcome assessment tools, and follow‐up duration introduced notable confounding factors. Third, publication bias cannot be excluded, as smaller studies with non‐significant findings are less likely to be published, especially in subgroups with limited data. Additionally, not all included articles distinguished between keloids and hypertrophic scars before and after treatment, which may have interfered with synthesis and analyses.

Despite these limitations, our findings have important clinical implications. The combined intervention of cryotherapy plus intralesional steroids is most suitable for patients with scars refractory to steroid monotherapy or other conventional treatments, and keloid scars with a narrow base may be considered ideal candidates for this regimen. Conversely, the combined therapy may not be necessary for patients with access to laser plus steroid or surgical excision plus radiotherapy, as these alternative modalities offer comparable efficacy without the associated increased risk of adverse events.

## 5 Conclusion

Cryotherapy combined with intralesional steroid injection is a viable alternative to other first‐line treatments for keloids and hypertrophic scars. Clinical recommendations should be made cautiously, considering several key factors, including scar anatomical location, size, treatment cost, accessibility, treatment course duration, potential complications, and patient preferences. Future studies are warranted to standardize outcome measurement tools, optimize treatment protocols, and conduct long‐term follow‐ups to further validate the clinical utility and long‐term safety of this combined regimen.

## Author Contributions

X.F., X.Z., S.J. and X.H. performed the research. X.F., J.X., and Y.D. designed the research study. X.F., X.Z., and S.J. analyzed the data. X.F. and J.X. wrote the paper.

## Funding

This work was supported by SiChuan Science and Technology Program, 2023YFS0233.

## Ethics Statement

The authors have nothing to report.

## Consent

The authors have nothing to report.

## Conflicts of Interest

The authors declare no conflicts of interest.

## Data Availability

The datasets used and analyzed during the current review are available from the corresponding author on reasonable request.
